# Case of Poisoning by Oleum Gaultheriæ

**Published:** 1852-06

**Authors:** Thomas J. Gallaher

**Affiliations:** Pittsburgh, Pa.


### *Case of Poisoning by Oleum Gaultheriæ.* By Thomas J. Gallaher, M. D., of Pittsburgh, Pa.

A youth, named W. Wall, aged nine years, of a sanguine temperament, and easily affected with vomiting and purging by the reception of indigestible articles into his stomach, was persuaded by some boys to swallow a large quantity of the oil of gaultheria. The quantity which he drank cannot be exactly ascertained; though from the statements of the boy himself and others who were with him, we are led to believe that there was at least half an ounce. This occurred on Friday evening, April 23d, 1852, at five o'clock, when his stomach was, comparatively, if not entirely empty. A short time after this, he returned home and ate some supper. After supper he commenced vomiting, and speedily ejected from the stomach all it contained. What he threw up was strongly scented with the oil of mountain tea. The parents thinking he had been eating some of the leaves of this plant, were not at first alarmed about him, but supposed as soon as the stomach was cleared of its contents, he would get better, as he had had like spells before from eating articles equally indigestible. Retching and vomiting, however, continued the whole of the ensuing night whenever anything, as a sup of water, was given to him. The following morning, April 24th, at ten o'clock, I was called to the case.

On arriving, I heard the history of the case, as above described, and further that the patient had had two copious evacuations from his bowels during the night, in both of which the odor of the oil was clearly perceptible. At this time the irritability of the stomach was great, everything received into it being immediately thrown off. The patient complained of very severe and constant pain in the epigastric, and in the inferior part of the hypochondriac regions, which was greatly increased on pressure with the finger. His skin was hot; pulse 125 strokes per minute; mouth parched and dry, tongue dry, smooth and slightly swollen; and his speech rather indistinct, in consequence of the swollen condition of the tongue. The symptoms, however, which were peculiar in this case, were a most inordinate, uncontrolled appetite for food, notwithstanding the irritability of the stomach; a very slow, laborious, prolonged and peculiarly loud respiration, not stertorous; and a marked dulness of hearing, without impairment of the intellectual faculties. The boy was very restless, and there was a slight increase in the intenseness of the beating of the carotid arteries. His eyes were slightly injected with blood, though this is presumed to be owing to his constant cries for something to eat. His thirst was not as great comparatively as his hunger. He felt no pain in his head, and the temperature of this organ was not greater than that of his body. The pupils of his eyes dilated and contracted freely on the absence and the presence of light. The reasoning powers, so far as I could judge, remained unimpaired, and there was no stupor further than that produced by the dulness of hearing.

The opinion which I formed of the case was, that the patient was laboring under acute gastro-enteritis, from the direct contact of the poisonous oil with the gastro-enteric membrane, and that the nervous centres which govern the functions of hearing and respiration were so injured that they could not develop their nerve power with their accustomed facility.

Eight American leeches were applied to that part of the abdomen in which there was the greatest tenderness; and when these came off, a warm poultice of bread and milk was ordered to be placed over the same part, and to be renewed every fourth hour. A few grains of Hyd. Chlor. Mit. were rubbed up with a scruple of Sacch. Alb., and divided into several parts. One of these was ordered to be dusted upon his tongue every third hour; a teaspoonful of gum-water to be given immediately afterwards, to enable him to get it into the stomach. Every article in the form of food or drink was strictly prohibited, excepting a teaspoonful or two of cold water or gum water occasionally. A saline injection was administered, which was to be repeated every two hours, until it operated freely upon the bowels. I gave the Hyd. Chlor. Mit. in order that it might carry off, by its cathartic action, if it should remain upon the stomach, all the offending oil which might yet remain in the intestines. A portion of the patient's hair was removed from his head, and cold water, by means of a sponge, constantly applied to it.

Next morning, April 25, 8 o'clock. Patient worse. He has had but little vomiting since last visit, though has frequent desire to do so. His importunities for something to eat are piteous. Four ounces of blood, by means of cups, were taken from his epigastrium, and two from behind his ears. Powders discontinued. Poultices and injections continued.

Four o'clock same day. Better. Tongue moist, and abdomen less tender. Slept some during the day. A consultation was held at this hour. Patient pronounced improving. Ten o'clock P. M. Remains better, but still complains of great epigastric oppression and tenderness. His hearing has improved, breathing less laborious and prolonged. Cupped again on the abdomen to to the extent of five ounces. Cold to the head discontinued.

26th. Decidedly better. Hearing entirely restored. Pulse 100 per minute. Appetite still remains voracious. A fly blister was applied to the abdomen, to be followed by poultices. Larger quantity of gum water allowed.

27th. Still improving. Blister raised admirably. Slept soundly all night. Appetite less ravenous.

From this date the patient gradually improved, and in two weeks was entirely restored.

*Remarks.*—The principal object in reporting this case is to record the effects of the Oleum Gaultheriæ upon the human subject, when given in an over dose. But few instances of poisoning from this article are on record, and these are not accurately described, therefore the medicinal and toxicological properties of it are but imperfectly known. Writers describing gaultheria attribute to it only aromatic and slightly astringent properties. Instances are mentioned of death from its use in large doses, from its causing inflammation of the stomach.

The case above described clearly shows that this plant, or at least its essential oil, possesses more properties than are usually attributed to it. The peculiar symptoms which were produced by its specific action in this instance, were: 1st, great dulness of hearing, without a general disturbance of the cerebral functions; 2, a slow, laborious and loud respiration, not stertorous; 3, a most voracious appetite for food, accompanied by symptoms which indicated gastro-enteric inflammation. These symptoms plainly indicate that Oleum Gaultheriæ acts as a direct and powerful sedative, when given in an overdose, upon those nervous centres which govern the functions of hearing and respiration; and that it is a powerful irritant to the gastro-enteric mucous membrane, with which it comes in contact, lighting up in it intense inflammation, which inflammation was accompanied by a peculiarly morbid condition of the gastric nerves, as evidenced from the great appetite with which it is attended. It does not appear to exert any specific influence over the cerebro-spinal centres generally, but only upon a few situated in the medulla oblongata.

